# Sleep Quality in Spanish Adult Women: Associations with Sociodemographic Variables and Pre-Sleep Habits

**DOI:** 10.3390/healthcare14142234

**Published:** 2026-07-22

**Authors:** Andrés Vicente Marín Ferrandis, Valentina Micheluzzi, Michela Capoferri, Michela Piredda, Elena Sandri

**Affiliations:** 1 Faculty of Medicine and Health Sciences, Catholic University of Valencia San Vicente Mártir, c/Quevedo, 2, 46001 Valencia, Spain; andres.marin@mail.ucv.es (A.V.M.F.); elena.sandri@ucv.es (E.S.); 2 Clinical and Interventional Cardiology, Sassari University Hospital, 07100 Sassari, Italy; 3 Department of Medicine, Surgery and Pharmacy, University of Sassari, 07100 Sassari, Italy; 4 Department of Animal Production and Health, Veterinary Public Health and Food Science and Technology, Faculty of Veterinary Medicine, Institute of Biomedical Sciences, Cardenal Herrera-CEU University, 46115 Alfara del Patriarca, Spain; michela.capoferricapoferri@alumnos.uchceu.es; 5 Research Unit of Nursing Science, Department of Medicine and Surgery, Campus Bio-Medico di Roma University, 00128 Rome, Italy

**Keywords:** sleep quality, sleep hygiene, sociodemographic factors, habits, public health, Spain

## Abstract

**Highlights:**

**What are the main findings?**

- Sleep quality among Spanish adult women was mildly impaired overall, with frequent nocturnal awakenings but limited use of sleep medication and modest daytime dysfunction.
- Older age, lower income, and lower educational attainment were associated with poorer sleep outcomes, whereas health-related education was linked to more favourable sleep profiles.

**What are the implications of the main findings?**

- Public health strategies to improve women’s sleep should address socioeconomic and educational inequalities, rather than focusing only on individual bedtime habits.
- Gender-sensitive, non-pharmacological interventions combining sleep hygiene, stress management, and culturally adapted routines may help reduce sleep disparities in Mediterranean populations.

**Abstract:**

**Background/Objectives:** Sleep health is a key determinant of physical and psychological well-being, yet poor sleep quality remains common, particularly among women. Sociodemographic inequalities and culturally patterned pre-sleep behaviours may contribute to sleep disturbances, but their combined role in Mediterranean populations is still insufficiently understood. This study aimed to examine sleep quality and its associations with sociodemographic characteristics and pre-sleep habits among adult women living in Spain. **Methods:** A cross-sectional online survey was conducted among 785 Spanish adult women aged 18–64 years. Sleep quality was assessed using the Spanish version of the Pittsburgh Sleep Quality Index (PSQI). Additional questionnaire items collected information on sociodemographic variables and pre-sleep habits, including meal timing, stimulant consumption, physical activity, bedtime routines, screen exposure, and sleeping alone or with company. Data were analysed using chi-square tests, Mann–Whitney U tests, Kruskal–Wallis tests, Spearman’s correlations, and principal component analysis. **Results:** The mean global PSQI score was 7.5 (SD = 3.01), indicating mildly impaired sleep quality overall. Age showed the largest association with global PSQI score (ε^2^ = 0.052, *p* < 0.001), followed by income (ε^2^ = 0.010, *p* = 0.016). Frequent nocturnal awakenings were the most common sleep disturbance, whereas sleep medication use was uncommon. Older age and lower income were associated with poorer sleep outcomes, including shorter sleep duration, lower sleep efficiency, and higher global PSQI scores. Higher education and health-related training were generally associated with more favourable sleep profiles. Pre-sleep habits such as late dinners, stimulant use, and evening screen exposure were highly prevalent but showed weak and non-significant associations with global PSQI scores. **Conclusions:** In this cross-sectional sample, poorer sleep quality was associated with selected sociodemographic characteristics, whereas isolated pre-sleep behaviours showed weak or non-significant associations with global PSQI scores. Given the study design and non-probability sampling, these findings should be interpreted as associations and generalized with caution. They may inform future studies and support the development of gender-sensitive, non-pharmacological sleep-health strategies that consider socioeconomic and cultural context.

## 1. Introduction

Sleep is a fundamental biological process that supports cognitive function, metabolic regulation, and overall physiological balance. In recent years, a growing body of evidence has linked inadequate sleep duration and poor sleep quality to adverse health outcomes, including cardiometabolic disorders, neuropsychiatric conditions, and impaired immune function, which ultimately contribute to increased morbidity and mortality [1,2,3,4]. In this context, epidemiological studies highlight the substantial burden of poor sleep in both Spain and Europe: in a large population-based study conducted in Spain, 38.2% of adults were classified as having poor sleep quality according to the Pittsburgh Sleep Quality Index (PSQI), with women almost twice as likely as men to report poor sleep [5], while at the European level recent data indicate that insomnia symptoms affect roughly 20–25% of adults and that chronic insomnia disorder is present in around one in ten adults [6]. Considering these associations, sleep health, broadly defined to include duration, continuity, efficiency, timing, and perceived quality, has emerged as a critical determinant of public health. It is also important to recognize that the determinants of sleep quality are multifaceted, spanning biological, psychosocial, and behavioural dimensions. Research indicates that sleep disruptions may act both as early biomarkers and as independent contributors to disease progression [7,8,9].

The complexity of sleep health is underscored by gender differences, as women are consistently found to be at higher risk of poor sleep quality and insomnia than men. This vulnerability has been attributed to a constellation of factors, including hormonal fluctuations associated with the menstrual cycle and menopausal transition, caregiving responsibilities, and psychosocial stressors [10,11]. In the context of Mediterranean populations, such as those in Spain, additional cultural and behavioural factors come into play. The Mediterranean setting is relevant because sleep-related behaviors in Spain are embedded in specific cultural and environmental routines, including later evening meals, prolonged evening social and family activities, evening screen exposure, and climate-related factors such as warm temperatures and seasonal daylight variation [8,9]. These features do not make the context inherently adverse, but they may influence sleep timing, continuity, and perceived sleep quality differently from other settings [12,13]. Therefore, findings from this population may be most directly transferable to similar Mediterranean or Southern European contexts, while extrapolation to populations with different cultural routines, climates, and sleep–wake schedules should be made with caution [14]. Despite recognition of these influences, there remains a significant gap in population-based research specifically examining the interplay among sociodemographic factors, culturally specific pre-sleep habits, and overall sleep quality in Spanish adult women. From a behavioural standpoint, conceptual frameworks such as Spielman’s 3P model of insomnia and social–ecological models of sleep health underscore the role of modifiable pre-sleep habits as key perpetuating factors of poor sleep, embedded within broader social and cultural environments [15]. Building on this perspective, the present study focuses on culturally patterned pre-sleep behaviours in Spanish women living in a Mediterranean context. However, despite evidence documenting the high prevalence of poor sleep and its broad determinants, population-based research remains limited in examining how sociodemographic characteristics and culturally patterned pre-sleep behaviours jointly relate to sleep quality in adult women [5,8,16]. In response, this cross-sectional study evaluated sleep quality among Spanish women using the validated Spanish version of the Pittsburgh Sleep Quality Index (PSQI) [17], complemented by newly developed items assessing culturally relevant pre-sleep habits. This approach integrates established sleep assessment with context-specific behavioural measures, recognising that sleep disturbances emerge from interactions among behavioural, social, and cultural influences rather than from isolated lifestyle practices. Sleep quality in adult women is shaped by multiple biological, psychological, occupational, and social factors, which may interact with sociodemographic characteristics and daily routines [10,11,14]. Based on the variables assessed in this study, we hypothesized that poorer sleep quality would be associated with less favourable sociodemographic characteristics and with potentially disruptive pre-sleep behaviours, including late meals, stimulant consumption, evening screen exposure, and stimulating bedtime routines. Therefore, this cross-sectional study aimed to describe sleep quality among Spanish women aged 18–64 years and to explore its associations with selected sociodemographic characteristics and culturally patterned pre-sleep behaviours, with the goal of generating context-specific evidence to guide future research in similar Mediterranean settings [18,19].

## 2. Materials and Methods

### 2.1. Type of Study and Inclusion Criteria

A cross-sectional study was performed in a sample of Spanish women aged 18–64 years. Participants were considered eligible if they did not present acute or chronic medical conditions known to impair sleep quality and were not undergoing temporary situations that could substantially alter their usual lifestyle, such as hospitalization or incarceration.

Health status was self-reported by the participants via an explicit checklist of medical diagnoses. To preserve the representative nature of a community-dwelling sample, highly prevalent conditions such as obesity, mild-to-moderate anxiety, or depressive symptoms were not utilized as grounds for exclusion. Conversely, participants were excluded if they explicitly reported a formal physician diagnosis of severe clinical conditions known to fundamentally disrupt sleep architecture, including: diagnosed sleep disorders (e.g., severe obstructive sleep apnea or restless legs syndrome), severe neurological conditions, active oncology treatments, or acute transient disruptions such as hospitalization, major surgical interventions within the previous month, or incarceration.

### 2.2. Ethical Considerations

The present study was conducted in accordance with the ethical principles outlined in the Declaration of Helsinki [20] and was approved by the Research Ethics Committee of the Catholic University of Valencia (approval code UCV/2024-2025/060, dated 28 January 2025). Prior to completing the questionnaire, all participants were informed about the objectives of the study and were assured that their responses would remain anonymous and that all data would be analyzed in aggregate form. Informed consent was obtained from each participant, including their agreement to the publication of the study’s findings.

### 2.3. Instrument

Sleep quality was assessed using the validated Spanish version of the Pittsburgh Sleep Quality Index (PSQI) [17,21], a widely used instrument evaluating sleep over the previous month. The Spanish PSQI includes 19 items (10 questions) generating seven components—subjective sleep quality, sleep latency, sleep duration, habitual sleep efficiency, sleep disturbances, sleep medication use, and daytime dysfunction—each scored 0–3, yielding a global score of 0–21 (higher scores indicate poorer sleep quality).

The scoring and component allocation were executed strictly following the instructions of the Spanish validated version by Royuela [21]. While the sleep onset latency question natively includes categorical checkboxes in this Spanish version, bedtime and get-up times were collected as free-text entries and used mathematically to compute sleep efficiency, ensuring the psychometric integrity of the global score.

To capture context-specific habits potentially influencing sleep, seven additional qualitative questions were developed by the research team. Content validity was reviewed by an eight-member panel of university lecturers and clinical collaborators specializing in community health, lifestyle research, clinical psychology, and family medicine. Following this initial review, the questions were pilot-tested among 37 community-dwelling Spanish women aged 18–64 years to ensure demographic alignment with the main study cohort. Structured cognitive interviews were utilized to assess clarity and interpretation. Based on feedback from the pilot participants, minor modifications were made before final deployment: typical Spanish dietary examples were added to the meal-heaviness options to reduce subjectivity, and the examples of stimulant beverages were expanded to improve recall accuracy.

### 2.4. Data Collection

Participants were recruited via email and social media (primarily Instagram), supplemented by the research team’s professional and personal networks (e.g., LinkedIn, X/Twitter, WhatsApp, Facebook). A snowball sampling approach was used, with eligible respondents invited to share the survey link within their networks until the target sample size was reached. The minimum target sample size was mathematically estimated at 385 participants, assuming a 95% confidence level, a 5% margin of error, and a conservative 50% prevalence rate to maximize sample variance. Our active recruitment strategy yielded a final sample of 785 valid participants after screening, which comfortably exceeded the statistical requirement and reduced our final margin of error to approximately 3.5%, thereby enhancing the robust nature of the inferential tests.

The study followed STROBE guidelines [22], and data were collected between February and May 2025. This specific seasonal window was selected to capture a highly stable period of standard working and academic routines in Spain, minimizing the confounding effects of irregular vacation schedules or extreme summer temperatures on participants’ baseline sleep characteristics.

**Figure 1:**
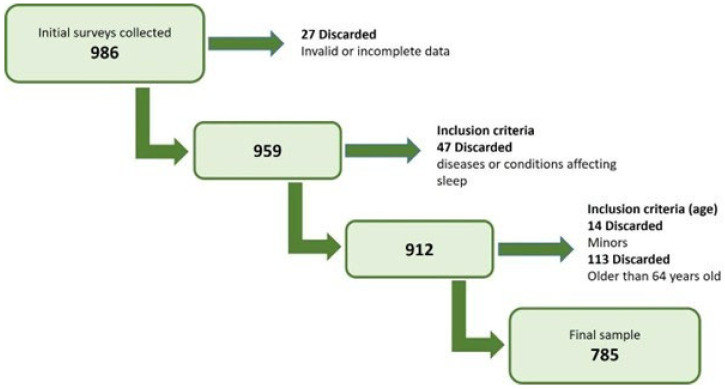
Diagram of the steps followed to obtain the final valid sample.

Because the online survey platform utilized only stores records upon successful final submission, the total number of initial page views and the specific dropout rate of unsubmitted questionnaires could not be tracked. A total of 986 completed questionnaires were successfully submitted. Following STROBE guidelines for cross-sectional studies, a rigorous data-cleaning protocol was applied as detailed in Figure 1: 27 questionnaires were immediately excluded due to incomplete or statistically incoherent data blocks; 47 respondents were excluded due to chronic or acute clinical conditions markedly altering baseline sleep architecture; and 127 participants were eliminated because they fell outside the targeted age spectrum (14 minors and 113 women older than 64 years). This multi-stage screening resulted in a final valid analytical sample of 785 participants.

### 2.5. Variables

#### 2.5.1. Socio-Demographic Variables

The survey collected information on participants’ sociodemographic characteristics, including age, place of residence, educational attainment, academic background, monthly income, and household composition. Age was categorized into four groups according to the classification proposed by Medley et al. [23]: young adults (18–22 years), early adulthood (23–34 years), early middle adulthood (35–44 years), and late middle adulthood (45–64 years). Educational attainment was dichotomized into basic education (no formal education, primary or secondary education, vocational training, or high school) and higher education (bachelor’s, master’s, or doctoral degree). Participants were also asked whether they had completed, or were currently enrolled in, a university program related to the health sciences. Monthly income was classified into three categories: low (≤EUR 2200/month), medium–high (>EUR 2200/month), and no response. Household composition was initially grouped as living alone or living with other people; participants living with others were further classified according to whether they resided in the parental home or in an independent household.

#### 2.5.2. Sleep Quality Variables

To facilitate data analysis and understand the specific habits of the sample, a two-step categorization process was conducted. First, raw sleep windows (bedtime and wake-up time) were grouped into categorical intervals to serve as independent descriptive variables representing typical Mediterranean schedules. Second, the seven official components of the PSQI were computed strictly following the standardized algorithms validated by Royuela & Macías [21], where bedtime and get-up time are utilized interactively to determine habitual sleep efficiency rather than receiving independent clinical scores. Table 1 reflects this complete framework, outlining both the descriptive schedule categories and the official psychometric scoring.

The original psychometric validation of the PSQI establishes a single binary clinical cut-off score of >5 to define poor sleep quality [14]. However, to provide greater descriptive granularity regarding the severity spectrum of sleep characteristics within a healthy, non-clinical cohort, the authors operationalized a three-tiered stratification framework based on the mathematical distribution of the global score. By dividing the total 0–21 scale into three symmetric intervals of 7 points each, global scores in this study were categorized into three progressive descriptive ranges: 0 to 7 indicated good sleep quality (encompassing optimal and subclinical lifestyle variations); 8 to 14 reflected sleep quality in need of improvement (moderate degradation); and 15 to 21 indicated poor sleep quality (severe clinical impairment). This author-defined, symmetric classification was adopted strictly for descriptive epidemiological segmentation to capture intermediate levels of sleep disturbance and prevent the over-pathologization of subclinical schedule shifts common in the Spanish population context.

#### 2.5.3. Habits and Routines That Might Influence Sleep Quality

To explore context-specific behavioral patterns, seven questions regarding pre-sleep habits were included. It must be explicitly emphasized that these items were treated purely as independent exploratory variables and not as a consolidated psychometric measurement instrument or summative scale. For descriptive and visualization purposes only, responses were categorized (Table 2) assigning a value of 0 to baseline behaviors and higher values to more active or stimulating variations, without assuming internal consistency or an underlying latent structure among them.

The theoretical standpoint guiding the formulation and scoring of these exploratory items is rooted in Spielman’s 3P model of insomnia, specifically focusing on modifiable perpetuating factors that trigger nocturnal psychophysiological arousal. Under this framework, items like the interval between the last meal and bedtime are evaluated not as nutritional markers, but as behavioral proxies for circadian phase-delay and thermal disruption common in Mediterranean lifestyle patterns. Similarly, the item tracking ‘pre-bedtime active disruptions’ (formerly translated as routines) explicitly measures secondary behaviors that prolong cognitive alertness or physical activation close to the sleep window (e.g., late-night household tasks or repetitive checking), meaning that higher scores mathematically represent higher behavioral stimulation rather than a lack of healthy sleep-hygiene unwinding practices.

### 2.6. Data Analysis

Initially, the data set was cleaned by removing erroneous or atypical entries (e.g., inaccurate responses and outliers). Normality was assessed using the Shapiro–Wilk test and Q–Q plots [24], showing that none of the variables were normally distributed. Accordingly, categorical variables were analysed with the chi-square test and reported as counts and percentages, while continuous variables were summarised as means and standard deviations. Given the large sample size (N = 785), the Law of Large Numbers and the Central Limit Theorem ensure that the sample mean remains a robust, stable, and unbiased population estimator despite the non-normal distribution of specific scores. Therefore, parameters in the tables are presented using means and standard deviations to ensure direct comparability with standard health sciences literature, while non-parametric inferential frameworks (Mann–Whitney U and Kruskal–Wallis tests) are applied to respect the rank-ordered nature of the data. Statistical significance was set at 0.05. To assess the clinical relevance and practical magnitude of the observed differences independently of the large sample size (N=785), non-parametric effect sizes were systematically calculated, reporting epsilon-squared ($\epsilon^{2}$) for the Kruskal–Wallis tests. Effect size magnitudes were interpreted following standard behavioral benchmarks: <0.01 as negligible, 0.01–0.06 as small, and 0.06–0.14 as moderate.

Spearman’s correlation coefficients were used to assess associations between sociodemographic variables and sleep-quality components, with significant correlations highlighted.

Principal component analysis (PCA) [25] was applied strictly as an unsupervised linear dimensionality reduction and exploratory visualization method. Because the exploratory pre-sleep habit variables and PSQI parameters are predominantly ordinal and non-normally distributed, the PCA was executed using a non-parametric Spearman rank-correlation matrix as its underlying computational input. This approach accounts for monotonic relationships among ordinal metrics without requiring multivariate normality. The number of retained components was determined based on the Kaiser criterion (eigenvalues >1.0) combined with a visual evaluation of the scree plot. In the resulting biplot, vector length indicates each variable’s contribution to the dimensions, and vector direction reflects inter-variable correlations. Analyses were conducted in jamovi (v2.4.44), which also generated the PCA plot; additional visualisations were produced in Microsoft Excel.

## 3. Results

### 3.1. Sample Composition

After applying data-cleaning procedures and inclusion/exclusion criteria (Figure 1), the final sample comprised 785 women. Following the STROBE checklist for observational studies, Figure 1 details the definitive multi-stage screening process. Out of the 986 total questionnaires received at the platform baseline, an aggregate of 201 entries were systematically excluded based on pre-established eligibility criteria and data completeness validation, leaving a final valid sample of 785 adult women for statistical analysis.

### 3.2. Sample Description

Participants were women aged 18–64 years, (mean = 36.7, SD = 13.3 years) evenly distributed across the four age groups (Table 3). Most lived with others (90.2%), usually family members (80.6%). Over half reported medium–high income (55.2%) and higher education (61.9%), and 35.5% had a background in health-related studies.

### 3.3. Analysis of the Sample’s Sleep Variables

**Figure 2:**
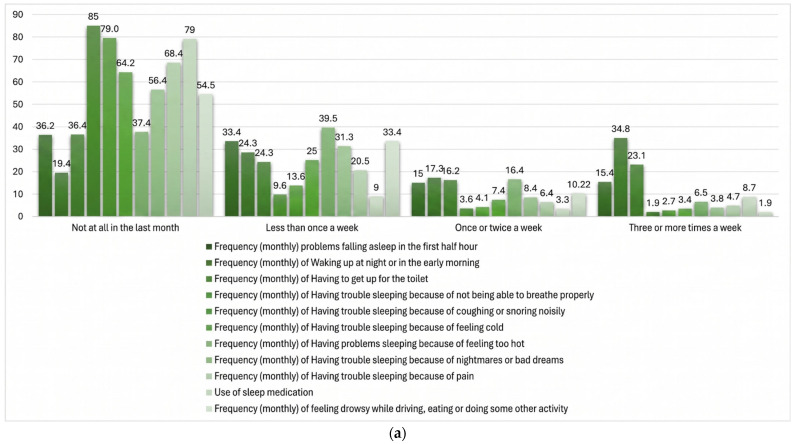

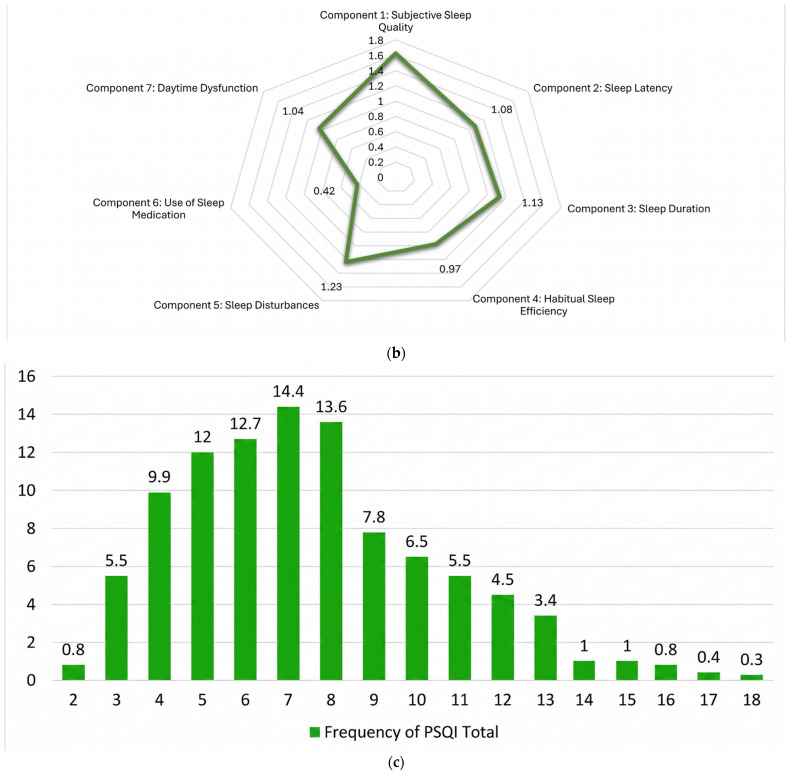
(**a**) Sleep disturbances (n = 785 adult women). Note: All numbers are expressed as percentages. (**b**) Distribution of the components of the PSQI sleep quality scale. (**c**) Relative distribution of the global Pittsburgh Sleep Quality Index (PSQI) scores (n = 785 adult women). Note: The *X*-axis represents the global PSQI score (0–21), and the *Y*-axis/data labels indicate the percentage (%) of the sample for each score.

Table 4 and Figure 2a–c summarize participants’ sleep habits and overall sleep quality. Most women went to bed between 11:00 p.m. and midnight and woke up between 6:00 and 8:00 a.m. In general, they reported falling asleep quickly: over half needed ≤15 min and about one-third 15–30 min. Self-perceived sleep quality was mostly favourable, with around 60% rating it as fairly or very good, and the majority reporting no or only occasional difficulties in performing daily activities the following day.

Figure 2a shows that the most frequent complaint was waking during the night or early morning, reported three or more times per week by about one-third of participants. Difficulties falling asleep within the first 30 min and getting up to use the toilet were also relatively common, whereas other disturbances (e.g., breathing problems, snoring, temperature discomfort, nightmares, pain) were mostly rare. Most women did not use sleep medication (85%) and seldom felt drowsy during activities such as driving or eating, indicating that serious disturbances and functional impairments were uncommon.

Table 4 and Figure 2b show the mean scores for the seven PSQI components. The highest scores were found for subjective sleep quality, sleep disturbances, sleep duration, and sleep latency, indicating challenges in perceived sleep quality, continuity, and time needed to fall asleep. Daytime dysfunction and habitual sleep efficiency were somewhat better, suggesting a limited impact on daily functioning, while use of sleep medication had the lowest score, confirming that most women rarely rely on pharmacological aids.

Fewer than 4% of women had very poor sleep quality (PSQI > 14). The complete distribution of these global scores across the sample is illustrated in Figure 2c, mapping the percentage of respondents against each achieved score.

### 3.4. Socio-Demographic Differences in Pittsburgh Sleep Quality Index (PSQI) and Its Components

Table 5a,b show that sleep quality differed significantly across several sociodemographic groups. PSQI global scores worsened with age, with late middle-aged women reporting the poorest overall sleep; younger women reported greater sleep latency and daytime dysfunction, whereas early/late middle age was associated with shorter sleep duration and lower sleep efficiency (*p* < 0.001). Low income was linked to poorer sleep (worse latency, more disturbances, higher daytime dysfunction) and a higher global PSQI score than medium–high income (*p* = 0.016). Living arrangement showed limited associations overall, although not living with family was related to longer latency and shorter sleep duration (*p* < 0.05). Higher education was generally associated with better latency, efficiency, and daytime functioning, and health-related education was consistently linked to better subjective sleep quality, longer duration, higher efficiency (all *p* < 0.001), and a slightly lower global PSQI score (*p* = 0.026).

Conversely, to maintain a balanced reporting of findings, it is critical to highlight that overall educational attainment (Studies) did not exhibit a statistically significant association with the global PSQI score (p=0.441, $\epsilon^{2}=0.004$). Furthermore, no significant differences were observed across income brackets or educational profiles regarding specific components such as sleep latency or daytime dysfunction, indicating that certain baseline sleep vulnerabilities remain uniform across the cohort regardless of socioeconomic advantages.

When exploring the practical magnitude of these sociodemographic variations on the global PSQI score, the calculated non-parametric effect sizes confirmed that age group exerted the most prominent layout ($\epsilon^{2}=0.0537$), followed by income level ($\epsilon^{2}=0.0105$). Conversely, despite achieving statistical significance due to the high sample power, the independent effect sizes for health-related training ($\epsilon^{2}=0.00632$) and overall educational attainment ($\epsilon^{2}=0.00407$) were small, indicating a heavily distributed impact across the cohort.

### 3.5. Correlation Matrix Between Sociodemographic Variables and Components of Sleep Quality

**Figure 3:**
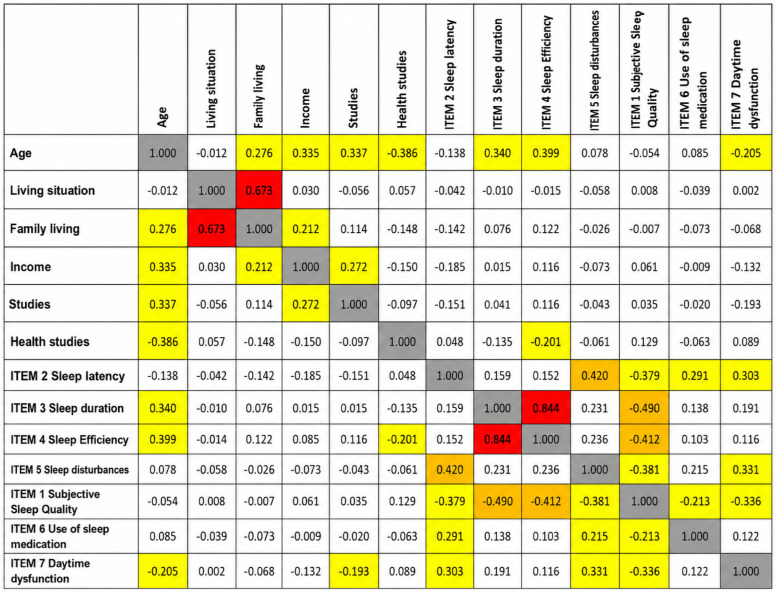
Spearman’s correlation matrix between sociodemographic variables and components of sleep quality. Legend: White cells = no correlation (0.00–0.19); yellow cells = low correlation (0.20–0.39); orange cells = moderate correlation (0.40–0.59); red cells = high correlation (≥0.60). Gray cells = diagonal self-correlations (r = 1.00).

Spearman’s correlations (Figure 3) were consistent with these findings, showing modest associations between age, income, education and several PSQI components, and strong correlations among core sleep parameters such as duration and efficiency.

### 3.6. Distribution of Pre-Sleep Habits of the Population

Table 6 summarizes pre-sleep habits in the sample. Most women slept with someone else and ate dinner 1–2 h before bedtime, usually a moderately heavy meal. The majority did not exercise close to bedtime, consumed stimulants mainly in the morning, and had no specific pre-bedtime active disruptions, although about one-quarter engaged in relaxing activities. Screen use before sleep was very common, with nearly 60% using screens within 30 min of bedtime and very few avoiding screens for at least two hours; the mean pre-sleep habits score (7.61, SD = 1.65) indicates room for improvement in sleep-supportive behaviours.

### 3.7. Analysis of the Relations Between Sample’s Pre-Sleep Habits and Sleep Quality

When comparing groups with good, intermediate, and poor sleep quality, none of the pre-sleep habits showed statistically significant differences (all *p* > 0.1; Table 7). Although some trends were observable (e.g., slightly lower stimulant use and more physical activity in women with better sleep), effect sizes were small and inconsistent. Consistent with these findings, correlation analyses indicated only weak associations between stimulant use and several indicators of poor sleep, whereas screen exposure before bedtime was essentially unrelated to PSQI components. Principal component analysis further showed that core sleep parameters (particularly duration, efficiency, and latency) accounted for most of the variance in sleep quality, while pre-sleep habits contributed only marginally.

### 3.8. Principal Component Analysis (PCA) Between Pre-Sleep Habit Variables and Sleep Quality Variables

**Figure 4:**
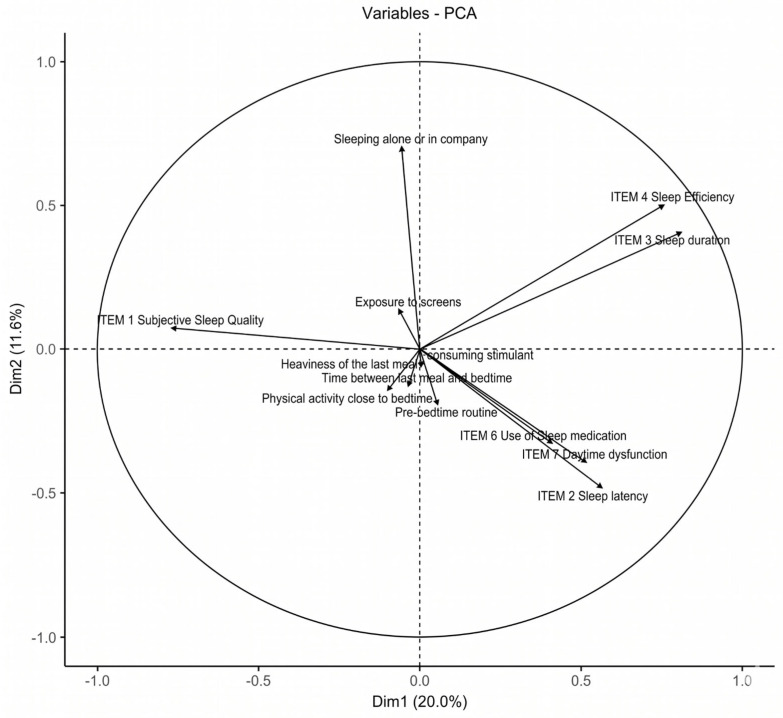
Principal Component Analysis (PCA) between pre-bed habit variables and sleep quality variables.

To mathematically map the spatial distribution of the parameters, an exploratory dimensionality reduction was executed based on a non-parametric Spearman matrix, utilizing the Kaiser criterion to retain the main components with eigenvalues >1.0 for low-dimensional mapping. The PCA in Figure 4 shows how the first two principal components explain a combined 31.6% of the variance (Dim1: 20.0%, Dim2: 11.6%), suggesting a moderately structured relationship among the variables. (The percentage of variance explained by each component is reflected in Table A1 of Appendix A).

ITEM 3 (Sleep duration) and ITEM 4 (Sleep efficiency) are strongly aligned and well represented on the right side of Dim1, indicating a shared positive contribution to sleep quality. In contrast, ITEM 2 (Sleep latency), ITEM 6 (Use of sleep medication), and ITEM 7 (Daytime dysfunction) cluster in the lower right quadrant and are oriented in the opposite direction from positive sleep indicators, reflecting their negative impact on restfulness and overall sleep quality.

Several pre-sleep behaviors, including pre-bedtime routine, physical activity close to bedtime, last meal timing, and stimulant consumption, are located near the origin, suggesting a limited contribution to the first two principal components or a more nuanced relationship. Only the fact of sleeping alone or in company seems to significantly affect the quality of sleep as the length of this arrow is considerable.

Altogether, the PCA highlights two dominant clusters: one grouping variables associated with poor sleep (e.g., medication use, long sleep latency), and the other aligned with indicators of healthy, efficient sleep. The role of pre-sleep habits appears multifactorial and requires further investigation beyond linear correlations.

## 4. Discussion

The present study examined sleep quality and related habits in adult women in Spain, using self-reported sleep parameters and the PSQI as the primary outcome. We focused on bed and wake times, sleep latency, subjective sleep quality, frequency of sleep disturbances, and the influence of sociodemographic characteristics and pre-sleep behaviours. This work is framed within a literature that highlights the role of both intrinsic sleep parameters and lifestyle factors, such as screen use and meal timing, in shaping sleep outcomes [26]. An important finding is that 44.8% of participants had a global PSQI score > 7, while 57.5% exceeded the conventional PSQI cut-off of >5 proposed by Buysse et al. [17]. This suggests a substantial burden of poor sleep quality among Spanish adult women. However, fewer than 4% of participants had very poor sleep quality, indicating that most sleep impairment in this sample was mild to moderate rather than severe. In this sample, associations with sleep quality appeared more consistent for selected sociodemographic variables than for isolated pre-sleep behaviours. However, because no multivariable model was performed to directly compare the relative contribution of these groups of variables, no hierarchy among determinants can be established.

Our findings partly diverge from previous studies reporting associations between pre-sleep habits, especially problematic smartphone use and delayed bedtimes due to evening screen exposure, and poorer sleep latency and efficiency [26,27,28]. In our sample, 59.0% of women used screens within 30 min of bedtime, but this behaviour did not show strong linear associations with global PSQI scores. This absence of statistically significant associations should not be interpreted as evidence that evening screen exposure or other pre-sleep behaviours have no influence on sleep quality. Rather, using the exploratory categorical items employed in this study, no consistent associations were observed within this sample. One possible explanation is the normalisation of these habits: when most individuals share similar behaviours, such as evening screen use or comparable meal timing, between-subject variability is limited, making statistical associations more difficult to detect. In addition, complex behavioural habits are difficult to capture through closed categorical questions. Their effects may depend on qualitative aspects not fully assessed here, such as the type and duration of screen use, nutritional composition of the evening meal, psychological arousal, stress, anxiety, workload, chronotype, and caregiving responsibilities. Therefore, the lack of significant differences between good and poor sleepers may reflect the limited granularity of the survey items rather than the absence of a true behavioural effect. The relatively regular sleep–wake schedule observed in this population, with bedtimes mainly between 23:00 and 00:00 and wake-up times between 6:00 and 8:00 a.m., may also have buffered the impact of potentially disruptive pre-sleep behaviours. Similar considerations may apply to stimulant use and evening dietary patterns, which did not show strong or statistically significant relationships with global PSQI scores in this sample, despite evidence from other settings [29,30].

From a behavioural standpoint, these findings can be interpreted considering Spielman’s 3P model and social–ecological models of sleep health. Pre-sleep habits are conceptualised as modifiable perpetuating factors of poor sleep; however, in this sample, the observed associations between individual pre-sleep behaviours and global PSQI scores were weak or inconsistent [15]. Given our large sample size (N = 785), several sociodemographic differences reached statistical significance. However, the associated non-parametric effect sizes fell within small-to-moderate thresholds (ranging from ϵ^2^ = 0.004 to ϵ^2^ = 0.054). Although these associations were statistically significant, their clinical relevance should be interpreted cautiously. Small differences in PSQI components may reach statistical significance in large samples without necessarily reflecting clinically meaningful differences in sleep quality at the individual level. Therefore, these findings should be interpreted as population-level associations rather than strong individual predictors. This is consistent with a social–ecological perspective in which sleep quality is shaped by multiple interacting behavioural, social, and contextual factors, rather than by isolated pre-sleep habits or single socioeconomic variables [14].

By contrast, our results clearly support the presence of socioeconomic and demographic gradients in sleep quality. In line with earlier work [31,32,33], lower income and lower educational attainment were associated with more sleep disturbances, longer sleep latency, reduced sleep efficiency, and higher global PSQI scores. Having a background in health-related studies was linked to more favourable sleep patterns, consistent with evidence that greater health literacy and awareness of behavioural risk factors promote healthier sleep practices [34]. These findings suggest that socioeconomic and educational factors shape women’s capacity to manage or compensate for potentially disruptive habits: those with higher income or more education, particularly in health sciences, may be better able to implement sleep-hygiene strategies, whereas women with lower socioeconomic status may be more vulnerable to the amplifying effects of financial stress, psychosocial burden, and constrained living environments. Concurrently, highlighting the non-significant boundaries of our data is equally vital to prevent reporting bias. The absolute level of general education showed no structural association with global sleep parameters. This lack of significance suggests that general academic literacy, isolated from specific health or biological training, is insufficient to modify deeply ingrained behavioral routines or counteract the socio-occupational stressors that trigger sleep fragmentation in adult women, demonstrating that negative results provide essential boundaries for targeted public health interventions.

Age also emerged as a key determinant of sleep quality. Younger women reported longer sleep latency and greater daytime dysfunction, possibly reflecting higher psychological stress and more irregular routines, whereas middle-aged and older women showed shorter sleep duration and lower efficiency, in keeping with known age-related changes in sleep architecture, hormonal status, and nocturnal awakenings. The progressive increase in global PSQI scores across age groups underscores the cumulative impact of sleep disruption across the life course. This life-course perspective is further illuminated by the sample’s mean global score of 7.5 (SD = 3.01), which sits on the exact baseline of our intermediate category (‘in need of improvement’). While evaluating this healthy population under a strict clinical binary threshold (>5) would brand a disproportionate majority of the sample as pathological cases, our customized, symmetric three-tiered approach highlights that while Spanish adult women show widespread subclinical sleep phase displacements, actual severe sleep degradation remains restricted to a very small subset (<4%). This underscores the descriptive utility of using mathematically balanced stratification intervals when evaluating non-clinical, community-dwelling populations. Living situation showed a subtler pattern: although differences were modest, women not living with family tended to report longer sleep latency and shorter sleep duration, supporting the view that cohabitation and family presence may provide emotional stability and more regular routines, in line with evidence on the protective role of social support for sleep health [35,36].

Correlation analyses and PCA indicate that intrinsic sleep parameters account for most variability in self-reported sleep quality. The first two components explained roughly one-third of the variance, defining a continuum from better sleep (higher duration and efficiency) to poorer sleep (greater latency, sleep medication use, and daytime dysfunction). Most pre-sleep behaviours clustered near the origin, suggesting minimal contribution to these main dimensions. A notable exception was sleeping alone versus with company, implying that the social context of sleep (e.g., co-sleeping arrangements) may modestly influence sleep quality [35,36].

Overall, socioeconomic gradients in sleep quality were clearly replicated, whereas specific pre-sleep behaviours showed a modest, context-dependent contribution. Intrinsic physiological factors and chronic stress likely play a larger role, potentially masking the effects of isolated pre-bedtime habits and driving key outcomes such as nocturnal awakenings, prolonged sleep latency, and daytime dysfunction.

### 4.1. Strengths and Limitations

Strengths of this study include the large, socioeconomically diverse sample of Spanish women and the use of the validated Spanish PSQI, enabling a detailed multidimensional assessment of sleep quality. Expert-validated, context-specific items on pre-sleep habits captured culturally relevant behaviours, and the combination of inferential analyses with PCA offered a broader view of how sleep parameters and habits cluster.

However, some limitations must be acknowledged. The cross-sectional design, which precludes causal inference and allows potential reverse causality, and reliance on self-reported data, with possible recall and social desirability bias. Furthermore, because data collection relied entirely on self-reported questionnaires without secondary verification through clinical records or medical examinations, the exact diagnostic severity of comorbidities (such as anxiety or chronic pain) could not be objectively quantified, introducing a potential source of classification bias standard in online epidemiological surveys. Snowball recruitment via social media and health-related networks may have favoured more health-conscious participants, limiting generalisability. Additionally, while the data collection period (February–May) effectively controlled for vacation-related routine disruptions and extreme summer heatwaves, the potential influence of shifting seasonal light patterns (photoperiod transition from winter to spring) on circadian alignment cannot be completely ruled out due to the cross-sectional design. Additionally, the ad hoc qualitative items utilized to measure pre-sleep habits may lack the psychological and contextual precision required to capture subtle behavioral variations. Since habits like bedtime routines and dietary timing are highly interconnected and non-linear, reducing them to rigid categorical brackets limits our ability to detect indirect or conditional associations with global PSQI scores. Additionally, while this study benefits from high statistical power due to its large cohort, the reliance on small-to-moderate effect sizes among the significant sociodemographic indicators means that the clinical predictability of these factors should be interpreted with caution when designing targeted individual treatments. Finally, while executing the exploratory PCA using a non-parametric Spearman matrix mitigated the non-normal, ordinal nature of our survey items, the first two components explained only a modest share of the cumulative variance (31.6%). This mathematical behavior confirms that these exploratory pre-sleep items do not possess a single, highly integrated structural cohesion, and implies that unmeasured latent constructs—such as occupational stress, chronic anxiety, genetic predisposition, or micro-environmental household factors—likely dominate the true underlying variance of sleep quality in this population. In addition, employment status, occupation, working hours, household responsibilities, type of dwelling, and rural/urban residence were not collected; future studies should include these variables to better contextualize socioeconomic differences in sleep quality. Finally, because this study was conducted in a Spanish Mediterranean context, the findings should be generalized cautiously to populations with substantially different cultural routines, climatic exposures, meal timing, and sleep–wake schedules.

### 4.2. Implications for Public Health and Future Directions

The strong links between age, income, education and sleep quality support targeted, gender-sensitive strategies to reduce sleep inequalities. In Spain, interventions should combine structural measures addressing social inequities with individual-level actions integrated into broader health promotion and education programmes [37,38]. Given the weak associations between single pre-sleep behaviours and PSQI scores, policies should prioritise multifactorial, non-pharmacological strategies—integrating sleep hygiene, stress management, diet and physical activity—delivered through community and primary care settings [8,9,39,40,41,42,43,44,45,46].

Future research should use longitudinal designs to clarify causal pathways and the effects of major life transitions (e.g., menopause, socioeconomic changes), and incorporate objective sleep measures alongside self-reports. Studies should also assess psychological determinants (stress, anxiety, mood disorders) and their interaction with sociodemographic and behavioural factors. Cross-cultural comparisons within Mediterranean settings could help isolate the influence of local norms and inform region-specific strategies. Finally, well-designed intervention trials combining educational and behavioural components, delivered through public health and primary care, are needed to develop effective, culturally tailored programmes that reduce gender- and socioeconomic-related sleep disparities.

## 5. Conclusions

This study describes sleep quality and pre-sleep habits among adult women in Spain, indicating that sleep reflects interacting biological, social, and behavioural determinants. Overall sleep was mildly impaired, with frequent nocturnal awakenings, limited use of sleep medication, and modest daytime impact. Socioeconomic and demographic gradients were consistent: older age, lower income, and lower educational attainment were associated with shorter, less efficient sleep, more disturbances, and higher global PSQI scores, whereas health-related training was linked to more favourable sleep profiles.

In contrast, common Mediterranean pre-sleep habits (late dinners, stimulant intake, evening screen exposure) were prevalent but showed weak, inconsistent associations with PSQI and explained little variance in sleep quality. These findings support multidimensional, gender-sensitive public health approaches prioritising socioeconomic inequalities and non-pharmacological strategies (sleep hygiene, stress management, culturally adapted routines), and offer a framework for future population-based research and targeted interventions.

## Figures and Tables

**Table 1:** Categorization of variables measuring sleep quality.

Parameter/PSQI Component	Category/Range	Role in Analysis/Analysis Type
**Exploratory Sleep Schedules** *(Analyzed purely as independent descriptive variables)*		
**Time to go to bed**	Before 10 p.m.	*Descriptive Variable*
	Between 10 p.m. and 11 p.m.	*(Used to calculate Component 4)*
	Between 11 p.m. and 12 a.m.	
	Later than 12 a.m.	
**Get up time**	Before 6 a.m.	*Descriptive Variable*
	Between 6 a.m. and 7 a.m.	*(Used to calculate Component 4)*
	Between 7 a.m. and 8 a.m.	
	After 8 a.m.	
**Standardized PSQI Components** *(Computed strictly following Royuela, 1997)*		
**Component 1: Subjective Sleep Quality**	Very Good/Fairly Good/Fairly Poor/Very Poor	0/1/2/3
**Component 2: Sleep Latency**	Score Sum: 0	0
*(Question 2 selection + Question 5a frequency)*	Score Sum: 1–2	1
	Score Sum: 3–4	2
	Score Sum: 5–6	3
**Component 3: Sleep Duration**	>7 h/6–7 h/5–6 h/<5 h	0/1/2/3
**Component 4: Habitual Sleep Efficiency**	>85%	0
*(Hours slept/Hours in bed x 100)*	75–84%	1
	65–74%	2
	<65%	3
**Component 5: Sleep Disturbances**	Total Sum: 0	0
*(Sum of scores from Q5b to Q5j)*	Total Sum: 1–9	1
	Total Sum: 10–18	2
	Total Sum: 19–27	3
**Component 6: Use of Sleep Medication**	Not at all/<Once a week/1–2 times a week/≥3 times a week	0/1/2/3
**Component 7: Daytime Dysfunction**	Score Sum: 0	0
*(Sum of Q8 + Q9)*	Score Sum: 1–2	1
	Score Sum: 3–4	2
	Score Sum: 5–6	3

**Table 2:** Categorisation of habits and routines that might influence sleep quality.

**Sleeping alone or in company**	**Categorisation**
Alone	0
With company	1
**Time elapsed between last meal and bedtime**	
<1 h	0
1–2 h	1
2–3 h	2
>3 h	3
**Heaviness of the last meal of the day**	
I don’t usually dine	0
Light (e.g., salad, soup, fruit)	1
Moderate (e.g., balanced plate with protein, carbohydrates and vegetables)	2
Heavy (e.g., high-fat, high-carbohydrate or large portions)	3
**Do you engage in physical activity close to bedtime?**	
I do not engage in physical activity before going to bed	0
Yes, >2 h before bedtime	1
Yes, 1–2 h before bedtime	2
Yes, <1 h before bedtime	3
**Do you consume any type of stimulant during the day (tea, coffee, energy drinks, caffeinated beverages, caffeine supplements)?**	
I do not use stimulant	0
Yes, in the morning	1
Yes, in the morning and in the afternoon	2
Yes, in the evening	3
**Do you have pre-bedtime active disruptions?**	
I don’t have a specific routine	0
Yes, I include relaxing activities (e.g., reading, meditating, listening to soft music)	1
Yes, I engage in stimulating activities (e.g., work, video games, intense exercise)	2
**Do you usually have exposure to screens before going to bed (mobile phone, computer, television)?**	
I do not expose myself to screens for at least 2 h before going to bed	0
Yes, 1–2 h before bedtime	1
Yes, 30–60 min before bedtime	2
Yes, <30 min before bedtime	3

**Table 3:** Sample and their socio-demographic characteristics (N = 785).

	Mean	SD
**Age (years)**	36.7	13.3
	**N**	**%**
Young (18–22)	149	19.0%
Early Adulthood (23–34)	215	27.4%
Early Middle Age (35–44)	206	26.2%
Late Middle Age (45–64)	215	27.4%
**Living situation**		
Alone	77	9.8%
With others	708	90.2%
**Family living**		
Without family	152	19.4%
With family	633	80.6%
**Income**		
No answer	97	12.4%
Low Income	255	32.5%
Medium–high Income	433	55.2%
**Education**		
Basic	299	38.1%
High	486	61.9%
**Health studies**		
No	506	64.5%
Yes	279	35.5%

**Table 4:** Sleep variables of the sample (n = 785 adult women).

Variable/PSQI Component	Category/Statistic	N/Mean	%/SD
Time to go to bed	Before 10 p.m.	60	7.60%
	Between 10 p.m. and 11 p.m.	230	29.30%
	Between 11 p.m. and 12 a.m.	335	42.70%
	Later than 12 a.m.	160	20.40%
Get up time	Before 6 a.m.	59	7.50%
	Between 6 a.m. and 7 a.m.	312	39.70%
	Between 7 a.m. and 8 a.m.	294	37.50%
	After 8 a.m.	120	15.30%
Time needed to fall asleep	≤15 min	416	53.00%
	16–30 min	252	32.10%
	31–60 min	96	12.20%
	≥60 min	21	2.70%
Subjective Sleep Quality	Very Poor	38	4.80%
	Fairly Poor	265	33.80%
	Fairly Good	430	54.80%
	Very Good	52	6.60%
Problems in getting in the mood for activity	No problem	428	54.50%
	Only a slight problem	262	33.40%
	A problem	80	10.20%
	A serious problem	15	1.90%
Component 1: Subjective Sleep Quality	Continuous Score (Mean/SD)	1.63	0.68
Component 2: Sleep Latency	Continuous Score (Mean/SD)	1.08	0.95
Component 3: Sleep Duration	Continuous Score (Mean/SD)	1.13	0.99
Component 4: Habitual Sleep Efficiency	Continuous Score (Mean/SD)	0.97	1.1
Component 5: Sleep Disturbances	Continuous Score (Mean/SD)	1.23	0.5
Component 6: Use of Sleep Medication	Continuous Score (Mean/SD)	0.42	0.91
Component 7: Daytime Dysfunction	Continuous Score (Mean/SD)	1.04	0.89
PSQI TOTAL	Continuous Score (Mean/SD)	7.5	3.01

**Table 5:** (**a**) Socio-demographic differences in Pittsburgh Sleep Quality Index (PSQI) and its components (n = 785 adult women). (**b**). Socio-demographic differences in Pittsburgh Sleep Quality Index (PSQI) and its components (n = 785 adult women).

(**a**)																
		**Total Sample**	**Age**										**Income**			
			**Young**		**Early Adulthood**		**Early Middle Age**		**Late Middle Age**				**Low Income**	**Medium–High Income**		
**PSQI Scale Scores**		**Mean (SD)**	**Mean (SD)**		**Mean (SD)**		**Mean (SD)**		**Mean (SD)**		***p*-Value** $	**ε^2^**	**Mean (SD)**	**Mean (SD)**	***p*** **-Value &**	**ε^2^**
Component 1: Subjective Sleep Quality		1.63 (0.68)	1.73 (0.66)		1.62 (0.68)		1.57 (0.69)		1.63 (0.68)		0.244	0.005	1.49 (0.70)	1.69 (0.66)	0.001 *	0.018
Component 2: Sleep Latency		1.08 (0.95)	1.36 (0.92)		1.09 (0.95)		0.96 (0.98)		0.98 (0.91)		<0.001 *	0.026	1.30 (1.00)	0.91 (0.88)	<0.001 *	0.037
Component 3: Sleep Duration		1.13 (0.99)	0.79 (0.67)		0.71 (0.81)		1.43 (1.04)		1.50 (1.04)		<0.001 *	0.140	1.22 (0.99)	1.13 (1.03)	0.068	0.007
Component 4: Habitual Sleep Efficiency		0.97 (1.10)	0.29 (0.61)		0.61 (0.89)		1.44 (1.17)		1.35 (1.13)		<0.001 *	0.180	1.11 (1.12)	1.00 (1.11)	<0.001 *	0.038
Component 5: Sleep Disturbances		1.23 (0.50)	1.12 (0.43)		1.25 (0.48)		1.25 (0.52)		1.27 (0.52		0.027	0.012	1.33 (0.57)	1.18 (0.44)	0.003 *	0.015
Component 6: Use of Sleep Medication		0.42 (0.91)	0.42 (0.89)		0.33 (0.79)		0.29 (0.82)		0.62 (1.08)		<0.001 *	0.027	0.46 (0.97)	0.39 (0.88)	0.907	0.000
Component 7: Daytime Dysfunction		1.04 (0.89)	1.38 (0.88)		1.03 (0.86)		1.01 (0.91)		0.83 (0.85)		<0.001 *	0.046	1.14 (0.95)	0.93 (0.84)	0.001 *	0.017
**PSQI TOTAL**		**7.50 (3.01)**	7.09 (2.39)		6.64 (2.66)		7.97 (3.35)		8.20 (3.13)		<0.001*	0.052	8.04 (3.26)	7.25 (2.90)	0.016 *	0.010
(**b**)																
	**Living Situation**				**Family living**				**Studies**				**Health Studies**			
	**Alone**	**With Others**			**Without Family**	**With Family**			**Basic Education**	**High Education**			**Yes**	**No**		
**PSQI Scale Scores**	**Mean (SD)**	**Mean (SD)**	***p*** **-Value &**	**ε^2^**	**Mean (SD)**	**Mean (SD)**	***p*** **-Value &**	**ε^2^**	**Mean (SD)**	**Mean (SD)**	***p*** **-Value &**	**ε^2^**	**Mean (SD)**	**Mean (SD)**	***p*** **-Value &**	**ε^2^**
Component 1: Subjective Sleep Quality	1.62 (0.73)	1.63 (0.68)	0.824	0.000	1.64 (0.69)	1.63 (0.68)	0.835	0.000	1.60 (0.69)	1.65 (0.67)	0.324	0.001	1.57 (0.68)	1.75 (0.66)	<0.001 *	0.017
Component 2: Sleep Latency	1.19 (0.96)	1.07 (0.95)	0.238	0.002	1.33 (0.91)	1.02 (0.95)	<0.001 *	0.020	1.26 (0.96)	0.97 (0.93)	<0.001 *	0.023	1.06 (0.99)	1.12 (0.87)	0.18	0.002
Component 3: Sleep Duration	1.17 (0.99)	1.13 (0.99)	0.771	0.000	0.98 (0.90)	1.17 (1.00)	0.033	0.006	1.08 (0.93)	1.16 (1.02)	0.251	0.002	1.23 (1.01)	0.95 (0.91)	<0.001 *	0.018
Component 4: Habitual Sleep Efficiency	1.01 (1.13)	0.97 (1.10)	0.685	0.000	0.70 (0.99)	1.03 (1.12)	<0.001 *	0.015	0.82 (1.09)	1.06 (1.10)	0.001 *	0.014	1.12 (1.12)	0.69 (1.02)	<0.001 *	0.040
Component 5: Sleep Disturbances	1.32 (0.57)	1.22 (0.49)	0.104	0.003	1.26 (0.54)	1.22 (0.49)	0.464	0.001	1.25 (0.53)	1.22 (0.48)	0.233	0.002	1.25 (0.52)	1.19 (0.45)	0.086	0.004
Component 6: Use of Sleep Medication	0.51 (0.97)	0.41 (0.91)	0.275	0.002	0.51 (0.94)	0.40 (0.91)	0.041	0.005	0.44 (0.92)	0.41 (0.91)	0.584	0.000	0.46 (0.95)	0.35 (0.84)	0.08	0.004
Component 7: Daytime Dysfunction	1.03 (0.86)	1.04 (0.90)	0.946	0.000	1.16 (0.90)	1.01 (0.89)	0.057	0.005	1.25 (0.89)	0.91 (0.87)	<0.001 *	0.037	0.98 (0.88)	1.14 (0.91)	0.013 *	0.008
**PSQI TOTAL**	7.86 (2.89)	7.46 (3.02)	0.196	0.002	7.57 (2.71)	7.48 (3.08)	0.513	0.001	7.70 (2.89)	7.38 (3.07)	0.075	0.004	7.67 (3.10)	7.19 (2.81)	0.026	0.006

$ Kruskal–Wallis test; & Mann–Whitney test; * significant differences.

**Table 6:** Distribution of pre-sleep habits of the population.

	N	%
**Sleeping alone or in company**		
Alone	293	37.3%
With company	492	62.7%
**Time elapsed between last meal and bedtime**		
<1 h	131	16.7%
1–2 h	418	53.2%
2–3 h	191	24.3%
>3 h	45	5.7%
**Degree of heaviness of the last meal of the day**		
I don’t usually dine	19	2.4%
Light (e.g., salad, soup, fruit)	205	26.1%
Moderate (e.g., balanced plate with protein, carbohydrates and vegetables)	501	63.8%
Heavy (e.g., high-fat, high-carbohydrate or large portions)	60	7.6%
**Engage in physical activity close to bedtime**		
I do not engage in physical activity before going to bed	614	78.2%
Yes, >2 h before bedtime	119	15.2%
Yes, 1–2 h before bedtime	43	5.5%
Yes, <1 h before bedtime	9	1.1%
**Consuming any type of stimulant during the day (tea, coffee, energy drinks, caffeinated beverages, caffeine supplements)**		
No stimulant use	202	25.7%
Yes, in the morning	382	48.7%
Yes, in the morning and in the afternoon	199	25.4%
Yes, in the evening	2	0.3%
**Pre-bedtime active disruptions**		
I don’t have a specific routine	552	70.3%
Yes, I include relaxing activities (e.g., reading, meditating, listening to soft music)	210	26.8%
Yes, I engage in stimulating activities (e.g., work, video games, intense exercise)	23	2.9%
**Exposure to screens before going to bed (mobile phone, computer, television)**		
I do not expose myself to screens for at least 2 h before going to bed	25	3.2%
Yes, 1–2 h before bedtime	101	12.9%
Yes, 30–60 min before bedtime	196	25.0%
Yes, <30 min before bedtime	463	59.0%
	**Mean**	**SD**
Habits before Sleep Total	7.61	1.65

**Table 7:** Relationship between pre-sleep habits and sleep quality (n = 785 adult women).

Sleep Quality	Good	Need of Improvement	Bad	
	Mean (SD)	Mean (SD)	Mean (SD)	*p*-Value $
Sleeping alone or in company	0.62 (0.49)	0.65 (0.48)	0.58 (0.51)	0.643
Time elapsed between last meal and bedtime	1.19 (0.78)	1.19 (0.79)	1.11 (0.57)	0.933
Degree of heaviness of the last meal of the day	1.76 (0.60)	1.79 (0.62)	1.58 (0.90)	0.232
Engaging in physical activity close to bedtime	0.32 (0.63)	0.27 (0.62)	0.11 (0.32)	0.146
Consuming any type of stimulant during the day (tea, coffee, energy drinks, caffeinated beverages, caffeine supplements)	0.99 (0.62)	1.01 (0.73)	1.16 (0.60)	0.553
Pre-bedtime active disruptions	0.33 (0.52)	0.33 (0.54)	0.37 (0.60)	0.961
Exposure to screens before going to bed (smartphone, computer, television)	2.44 (0.82)	2.35 (0.84)	2.32 (0.89)	0.227
Habits before Sleep Total	7.63 (1.60)	7.59 (1.69)	7.21 (1.87)	0.807

$ Kruskal–Wallis test.

## Data Availability

The data supporting the findings of this study are available from the corresponding author upon reasonable request due to privacy.

## Abbreviations

The following abbreviations are used in this manuscript:

AI	Artificial Intelligence
PCA	Principal Component Analysis
PSQI	Pittsburgh Sleep Quality Index
SD	Standard Deviation
STROBE	Strengthening the Reporting of Observational Studies in Epidemiology

## Appendix A

**Table A1:** Eigenvalues of the PCA between pre-bed habit variables and sleep quality variables (Components and percentage of variance explained).

Component	% of Variance
1	19.95%
2	11.56%
3	10.30%
4	8.23%
5	7.95%
6	7.69%
7	6.71%
8	6.34%
9	5.87%
10	5.54%
11	4.89%
12	3.78%
13	1.18%
